# Hysteria

**Published:** 1905-07-01

**Authors:** 


### HYSTERIA.

In a delightful clinical lecture [1] Dr. Hale White has collected most of the cardinal features of this protean malady. In the first place he notes the falsity of the name, which is a relic of Aristotelian pathology, the frequency of the disease in women leading, not unnaturally in the circumstances, to its connection with some disorder of the uterus. A like train of thought, we may observe in passing, led the Shakespearian age to speak of hysteria as "the Mother," no doubt from its commonly feminine associations. "Oh, how this mother swells up toward my heart!" says King Lear; "Hysterica passio, down, thou climbing sorrow, Thy element's below." There is little doubt, however, that hysteria has no connection whatever with the sexual organs; Lear was merely describing in vivid language the same "Globus hystericus" which was the subject of "A Brief Discourse of a Disease called the Suffocation of the Mother," written by a Dr. Jordan in 1603.

Without attempting a definition of hysteria, Dr. Hale White lays down certain rules which must govern the diagnosis. For a given state to be hysterical it must be such that "by no means of dissection, histological analysis, or chemical pathology would it be possible in the patient to demonstrate after death that the body was in any way diseased." Again, for a case to be one of hysteria the symptoms must not accurately correspond to any known clinical picture, for no two cases of hysteria are actually alike. There are several diseases which have no known morbid anatomy, such as paralysis agitans, epilepsy, and some neuralgias, yet they cannot be classed as hysterical, for the clinical picture is too constant, and one case of paralysis agitans resembles another within narrow limits. Another criterion is that the whole character of the patient and the symptoms must indicate to the observer a lack of will power; the condition cannot be better described than in the words of Sir James Paget, quoted by the author. The patient says she "cannot," and her friends, not grasping what hysteria is, say "she will not"; and the doctor who does grasp what it is, says "she cannot will." This deficiency in will-power is a constant feature of hysterical states and supplies an explanation of such miraculous cures as those of Lourdes; for it inclines the patient to pander to the exaggerated self-consciousness which is an equally sure accompaniment of the condition, and, by the mutual interaction of these characteristics, symptoms which a healthy person would disregard are at first nursed and magnified, and very soon utilised as a means of invoking the attention and sympathy of others. The value of the pilgrimage to Lourdes and other such places is purely suggestive. The expectation of recovery provides the will with a stimulus sufficient to achieve the hitherto impossible task of throwing off the inveterate regard for symptoms in themselves insignificant.

As regards an explanation of the phenomena of hysteria, Dr. Hale White accepts with reservations the hypothesis of Myers, that in hysteria the supraliminal consciousness—the ordinary consciousness of self to be found in healthy individuals—is more or less in abeyance, and the subliminal self, whose mental processes are unperceived by the healthy, attains a morbid prominence. This argument is favoured by the fact that a patient subject to functional anæsthesia is not conscious that she cannot feel until it is pointed out to her, arguing that her supra-liminal consciousness, which should acquaint her of such a bodily alteration, is functionally inactive, but that her subliminal self is active to a sufficient degree to prevent injury to the insensitive surface, for the hysterically anæsthetic never injure themselves unawares in the way that is common in organic disorders of sensation such as syringo-myelia. That this relic of self-protection is really sub-liminal seems proved by an experiment quoted from Myers. A girl with an hysterical contraction of the visual field was very prone to hysterical fits, which might always be induced by the sight of a mouse. A stuffed mouse was slowly brought into the field of vision and was found to determine a fit long before it entered the central part of the field, in which alone her ordinary consciousness was able to perceive anything.

The author enters a much-needed word of warning against the too frequent confusion between hysteria and malingering, for to treat a hysterical girl as a malingerer is pure cruelty. Not infrequently such patients are the most thoughtful and self-sacrificing members of a family, and to assume that they do not believe in their symptoms is to inflict a most unjustifiable injury upon them.

With respect to motor symptoms it is noted that paraplegia and hemiplegia are common, but monoplegia excessively rare; the affected limbs do not waste, nor are their electrical reactions altered; in the case of hemiplegia the face is hardly ever affected, nor any of the cranial nerves, unless the recurrent laryngeal be considered a cranial nerve. The motor affection may be spasmodic as well as flaccid, as is instanced by the hysterical epileptic fit, by rapid respiration and barking coughs. The sensory symptoms are remarkable for their unorthodox limits, for they never follow accurately the anatomical distributions of nerves. For instance, in the case of anæsthesia of a limb, the upper limit is generally marked by a horizontal line straight round it. When the sight is affected the variety is always a general amblyopia, not the hemianopia of cerebral disease. Visceral symptoms are frequent, and often inexplicable. A patient has been known to vomit in ten minutes an enema coloured with methylene blue to prevent deception, although she was carefully observed throughout. Hysterical hæmatemesis is an undoubted fact, as is hysterical fever, though the author thinks that most such heightenings of temperature depend upon friction, or some fraudulent manipulation of the thermometer.

The treatment recommended is in the first place removal from the sympathy of friends; also, abundance of good food, and a draught of valerian and assafœtida, which last probably acts by suggestion. The Weir-Mitchell treatment is occasionally useful, but is often tried in most unsuitable cases. "The ideal case for Weir-Mitchell treatment is the wasted girl, the patient who has slowly wasted to a shadow from constant vomiting and inability to take food. If she is properly treated by the Weir-Mitchell form of treatment she will be well in about six weeks." This method is of no use when the patient is drifting towards definite mental disease, nor if the patient is very fat, nor, in the author's opinion, in cases of paralysis whether of sensation or motion. In further insisting on the difference between true hysteria and malingering, Dr. Hale White mentions the case of a girl of good position who was the subject of an extraordinary rash over nearly the whole of the trunk and limbs. The actual mode of causation was never discovered, but it was ascertained that by encasing a limb in plaster of Paris it was possible to exclude the enveloped limb from the area of eruption. It was quite clear that the girl was a malingerer pure and simple.

[1] Clin. Jour., June 7, 1905.

